# Central Effects of Beta-Blockers May Be Due to Nitric Oxide and Hydrogen Peroxide Release Independently of Their Ability to Cross the Blood-Brain Barrier

**DOI:** 10.3389/fnins.2019.00033

**Published:** 2019-01-31

**Authors:** Claire Laurens, Anne Abot, Alain Delarue, Claude Knauf

**Affiliations:** ^1^Pierre Fabre Dermatologie, Lavaur, France; ^2^Enterosys SAS, Prologue Biotech, Toulouse, France; ^3^INSERM U1220 Institut de Recherche en Santé Digestive, CHU Purpan, Université Toulouse III Paul Sabatier, Toulouse, France

**Keywords:** beta-blockers, propranolol, blood-brain barrier, infantile hemangioma, reactive oxygen species

## Abstract

Propranolol is the first-line treatment for infants suffering from infantile hemangioma. Recently, some authors raised the question of potential neurologic side effects of propranolol due to its lipophilic nature and thus its ability to passively cross the blood-brain barrier (BBB) and accumulate into the brain. Hydrophilic beta-blockers, such as atenolol and nadolol, where therefore introduced in clinical practice. In addition to their classical mode of action in the brain, circulating factors may modulate the release of reactive oxygen/nitrogen species (ROS/RNS) from endothelial cells that compose the BBB without entering the brain. Due to their high capacity to diffuse across membranes, ROS/RNS can reach neurons and modify their activity. The aim of this study was to investigate other mechanisms of actions in which these molecules may display a central effect without actually crossing the BBB. We first performed an oral treatment in mice to measure the accumulation of propranolol, atenolol and nadolol in different brain regions *in vivo*. We then evaluated the ability of these molecules to induce the release of nitric oxide (NO) and hydrogen peroxide (H_2_O_2_) *ex vivo* in the hypothalamus. As expected, propranolol is able to cross the BBB and is found in brain tissue in higher amounts than atenolol and nadolol. However, all of these beta-blockers are able to induce the secretion of signaling molecules (i.e., NO and/or H_2_O_2_) in the hypothalamus, independently of their ability to cross the BBB, deciphering a new potential deleterious impact of hydrophilic beta-blockers in the brain.

## Introduction

Since its discovery in 1960 by J. W. Black, the non-selective beta-blocker propranolol has been widely used in the treatment of hypertension, tachycardia and other cardiac disorders. More recently, propranolol has been introduced as first-line therapy for infantile hemangiomas, the most common soft-tissue tumors of childhood (Leaute-Labreze et al., 2015).

As propranolol is a lipophilic molecule, its use in infants raised the question of its ability to passively cross the blood-brain barrier (BBB) and directly activate adrenoreceptors on neuronal cells, and to subsequently impact the neurologic development of the child. Even if many reports have documented that propranolol treatment during infancy does not alter further brain development when compared to child and adolescents from the general population examined several years after cessation of treatment (Moyakine et al., 2016, 2017; Gonzalez-Llorente et al., 2017; Mahon et al., 2018), other beta-blockers with hydrophilic properties have been here or there introduced in the treatment of infantile hemangiomas. The most commonly used molecules are nadolol and atenolol, which display hydrophilic properties and are therefore suggested to be unable to cross the BBB (Neil-Dwyer et al., 1981; Dahlof and Dimenas, 1990) and thus potentially decrease the risk to induce deleterious central effects (de Graaf et al., 2013; Pope et al., 2013; Tasani et al., 2017; Alexopoulos et al., 2018). However, these assumptions are based on biophysical characteristics of these molecules, and data regarding long-term safety are clearly lacking.

Alternative mechanisms in which some molecules are able to induce signaling pathways in the brain without crossing the blood-brain barrier have been described. One mechanism, that requires diffusible factors between two partners, implies central neurons and endothelial cells located in vascular wall. Indeed, brain endothelial cells have the capacity to produce second messengers such as nitric oxide (NO) to control the release of neurotransmitters (Knauf et al., 2001). This is especially the case in the mediobasal hypothalamus that includes the median eminence, a circumventricular organ where the BBB is physiologically absent (Rodriguez et al., 2005, 2010; Horsburgh and Massoud, 2013). For example, circulating hormones like estrogen (Prevot et al., 1999) or apelin (Duparc et al., 2011) induce endothelial NO release, with consequences on physiological functions, such as ovulation and glucose homeostasis, respectively. Hydrogen peroxide (H_2_O_2_) is another way to induce a signaling pathway in this brain region. As previously described for NO signaling, high dose of apelin can modify the release of hypothalamic H_2_O_2_ that could participate to an over-activation of the sympathetic system leading to the development of a type 2 diabetic state (Drougard et al., 2014). Thus, some molecules can modulate the activity of neuronal cells despite the absence of passage through the BBB and of direct activation of specific receptors on these cells.

The aim of the present study was to investigate whether propranolol, atenolol and nadolol induce the secretion of signaling molecules (i.e., NO and H_2_O_2_) in the hypothalamus, independently of their ability to directly stimulate adrenoreceptors on neuronal cells.

## Methods

### Animals and Mass Spectrometry Analyses

All procedures are performed in accordance with the Directive 2010/63/UE recommendations and with French Veterinary Authorities agreement. *Ex vivo* design and procedures were approved by Ethical Committee (under protocol CEEA-122 2014-53). Eight weeks-old C57BL/6J male mice (*n* = 10/group, mean body weight = 25 g) were orally treated with either propranolol (3 mg/kg/day), atenolol (2 mg/kg/day) or nadolol (1 mg/kg/day) during 7 days, once a day for vehicle, nadolol and atenolol groups (at 8.00 am) and twice a day for propranolol group (at 8.00 am and 6.00 pm). Dose and administration scheme were selected to mimic therapeutic use of these molecules in infants suffering from infantile hemangioma. Mice were euthanized 1 h after the last gavage. Cortex, hypothalamus, cerebellum and brainstem were collected and immediately frozen in liquid nitrogen.

The concentrations of propranolol, atenolol and nadolol were determined after solid phase extraction followed by LC/ESI-MS/MS detection. Tissues were diluted in 9 ml of ultrapure water for 1 g of brain and homogenized by sonication over crushed ice. Atenolol-D7 and propranolol-D7 were used as internal standards. Dynamic concentration range was comprised between 1 and 8000 ng/ml for each compound. The chromatographic peaks for tested compound and internal standards were identified according to their retention times and MRM ion transitions and integrated by analytical software (MassLynx version 4.1, Waters).

### Nitric Oxide and Hydrogen Peroxyde *ex vivo* Amperometric Measurements

Calibration of the electrochemical sensor was performed as previously published (Duparc et al., 2011; Drougard et al., 2014; Fournel et al., 2017; Abot et al., 2018). After dissection, hypothalamus was washed in Krebs-Ringer bicarbonate/glucose buffer (pH 7.4) in an atmosphere of 95%O_2_–5%CO_2_ and then immersed in Eppendorf tubes containing the same medium. Spontaneous NO or H_2_O_2_ release was measured at 37°C for 10 min by using either a NO-specific (ISO-NOPF, World Precision Instruments) or a H_2_O_2_-specific (ISO-HPO, World Precision Instruments) amperometric probe implanted in the hypothalamus. Ten micro liter saline solution (vehicle) was injected directly in the survival medium. After 10 min of record, 10 μl beta-blocker solution at increasing concentration was injected (final concentrations: 50 and 250 ng/ml). These concentrations were chosen to mimic plasma concentration of these molecules after an oral therapeutic dose in humans (i.e., approximately 50 ng/ml for propranolol and nadolol, and 250 ng/ml for atenolol). The concentration of NO or H_2_O_2_ gas in solution was measured in real-time (TBR1025, World Precision Instruments). DataTrax2 software (World Precision Instruments) performed data acquisition. Data are expressed as delta variation of NO or H_2_O_2_ release from basal.

### Statistical Analyses

All statistical analyses were performed using GraphPad Prism 5.0 for Windows (GraphPad Software Inc., San Diego, CA, United States). Two-way ANOVA and Bonferroni’s *post hoc* tests when used when appropriate. All values are presented as mean ± SEM. Statistical significance was set at *p* < 0.05.

## Results

### Propranolol, Atenolol, and Nadolol Concentration in the Brain

**FIGURE 1:**
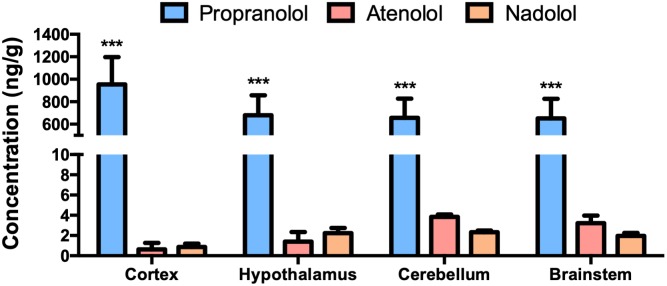
Accumulation of propranolol, atenolol, and nadolol in different brain regions after a chronic oral treatment in mice. Concentration of propranolol, atenolol, and nadolol measured in the cortex, hypothalamus, brainstem, and cerebellum after 1 week of daily oral treatment in mice; *n* = 10/group; ^∗∗∗^*p* < 0,001 vs. atenolol and nadolol.

We have first evaluated the capacity of the three beta-blockers to reach brain tissues (Figure 1). As previously described, propranolol is found at a high concentration in the brain after an oral treatment. In the same experimental conditions, the concentrations of nadolol and atenolol are lower in all brain regions, including a neurohemal structure such as the hypothalamus. This last result suggests that hydrophilic molecules cannot massively penetrate into the brain whether or not the BBB is leaky.

### Hypothalamic NO Release in Response to Beta-Blockers

**FIGURE 2:**
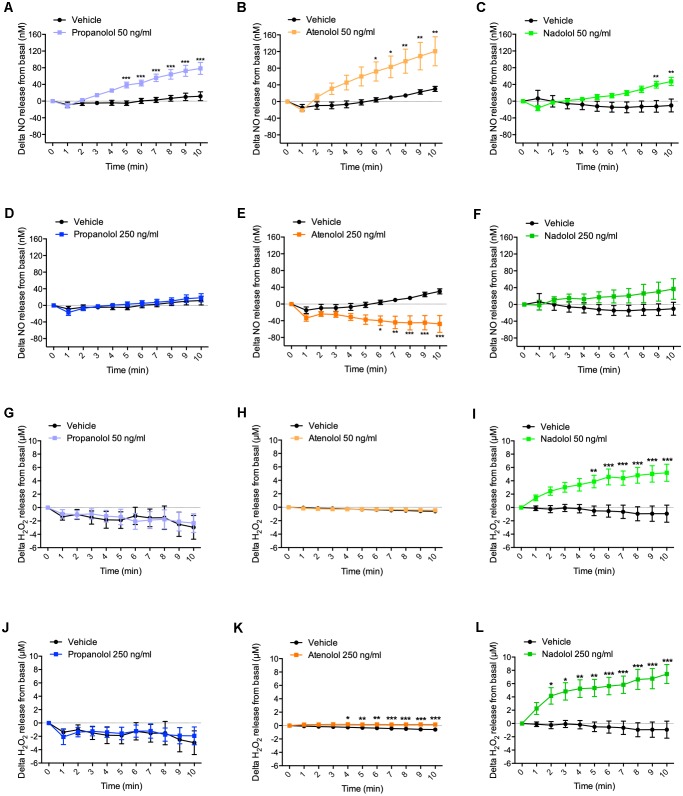
Hypothalamic NO and H_2_O_2_ release in response to propranolol, atenolol, and nadolol treatment. *Ex vivo* NO **(A–F)** and H_2_O_2_ **(G–L)** release from hypothalamus in response to propranolol **(A,D,G,J)**, atenolol **(B,E,H,K)**, or nadolol **(C,F,I,L)** at either low dose 50 ng/mL **(A–C,G–I)** or high dose 250 ng/mL **(D–F,J–L)**; *n* = 10/group; ^∗^*p* < 0,05, ^∗∗^*p* < 0,01, and ^∗∗∗^*p* < 0,001 vs. vehicle.

At the dose of 50 ng/ml, propranolol rapidly increased NO release from hypothalamus from 5 to 10 min (Figure 2A). Interestingly, atenolol was, at the same dose, also able to increase NO secretion from 6 to 10 min (Figure 2B), while delta variation of NO release in response to nadolol treatment was significantly induced after 9 min (Figure 2C). Taken together, these results show that, at a low dose, all three tested beta-blockers are able to induce NO release from hypothalamus.

When hypothalamus was treated with propranolol at a higher dose (i.e., 250 ng/ml), no significant variation in NO release was observed (Figure 2D). Surprisingly, atenolol displayed an opposite effect at the high dose compared to low dose treatment by significantly decreasing NO release after 6 min of treatment (Figure 2E). As observed for propranolol, nadolol treatment at this same dose did not induce any significant variation of NO release by the hypothalamus (Figure 2F).

These data indicate that lipophilic propranolol and hydrophilic atenolol and nadolol are all able to induce NO release from the hypothalamus at a low dose. Contrasting results observed at a higher dose suggest that alternative mechanisms may be activated to counterbalance the effects of massive NO release in the brain.

### Hypothalamic H_2_O_2_ Release in Response to Beta-Blockers

Treatment with a low dose of propranolol (i.e., 50 ng/ml) did not induce any significant variation of H_2_O_2_ release from the hypothalamus (Figure 2G). Similar results have been observed upon atenolol treatment (Figure 2H). However, in response to nadolol at a low dose, delta variation of H_2_O_2_ release from basal significantly increased from 5 min (Figure 2I).

At a higher dose of 250 ng/ml, no significant changes of H_2_O_2_ production were observed in response to propranolol treatment (Figure 2J), while both atenolol (Figure 2K) and nadolol (Figure 2L) treatment rapidly increased H_2_O_2_ release.

Altogether, these results clearly show that, while propranolol treatment does not induce H_2_O_2_ release from the hypothalamic explants at any dose, a high dose of atenolol or nadolol rapidly induce H_2_O_2_ release from the hypothalamus. The amplitude of H_2_O_2_ production is particularly high in response to nadolol treatment, either at a low or high dose.

## Discussion

We have shown that, despite the inability of hydrophilic beta-blockers to cross the BBB and thus to accumulate into the brain, all three beta-blockers tested in this study, either lipophilic or hydrophilic, were able to modulate the release of NO and/or H_2_O_2_ in the hypothalamus. As these small molecules can passively diffuse in brain tissue, they could have an impact on neuronal activity of central neurons. This could explain some of the potential deleterious effects of these molecules in the brain such as sleep disorders (de Graaf et al., 2013; Pope et al., 2013; Labreze et al., 2015; Leaute-Labreze et al., 2015; Randhawa et al., 2015; Ji et al., 2016) and in periphery such as hypoglycemia (Holland et al., 2010; Poterucha et al., 2015).

Despite its ability to accumulate into the brain, propranolol failed to increase the release of hypothalamic H_2_O_2_ that is usually associated with oxidative stress (Fisher-Wellman and Neufer, 2012; Angelova and Abramov, 2018). Little is known in the literature to explain the mode of action of propranolol to avoid H_2_O_2_ release. It has been shown that, in the skin of frog, propranolol is able to decrease water permeability induced by arginine vasotocin (AVT) (Ogushi et al., 2010; Saitoh et al., 2014). In this model, aquaporins are translocated at the membrane in response to AVT. We can speculate that propranolol, by acting on aquaporins translocation, could decrease the negative impact of H_2_O_2_, which is known to use aquaporins to cross cell membrane (Tamma et al., 2018).

The effect of atenolol on hypothalamic NO/H_2_O_2_ release is also unexpected. At low dose, atenolol induces NO release from hypothalamic explants, but at higher dose of 250 ng/ml, corresponding to plasma concentration measured after an oral administration at the therapeutic dose, it decreases NO release and increases H_2_O_2_ secretion. Even if this dual effect seems surprising, our group has previously demonstrated the existence of such physiological phenomenon in another physiological context. Indeed, apelin, an adipokine released by the adipose tissue, stimulates the release of hypothalamic NO at a low dose (Duparc et al., 2011), but inhibits NO release and stimulates H_2_O_2_ release at a high dose (Duparc et al., 2011; Drougard et al., 2014). In our experimental model, this dual effect can be explained by pharmacological and physiological hypotheses. For instance, the decrease of NO release could be due to its interaction with H_2_O_2_ to generate hydroxyl radical (Nappi and Vass, 1998).

In addition, we observed that nadolol induces both NO and H_2_O_2_ secretion either at a low or high dose. The low dose reproduces plasma concentration measured after an oral therapeutic dose of nadolol. This result demonstrates that the ability to cross the BBB is not mandatory to induce signaling in the brain, and thus further activation/inactivation of specific neuronal populations.

Finally, the magnitude of NO/H_2_O_2_ release in response to the different treatments also needs to be considered. Indeed, while a release in the range of pM to nM induces physiological effects such as signal transduction and neurotransmission (either beneficial or detrimental depending on targeted neurons), a release in the range of nM to μM induces deleterious effects such as oxidative stress and DNA damage leading to cellular dysfunction (Bredt, 1999; Mustafa et al., 2009). In this study we show that, at a concentration mimicking plasma concentration of the three beta-blockers after a therapeutic dose, propranolol induced a release of 80 nM of NO and no change in H_2_O_2_ production. In contrast, atenolol decreased NO release but increased H2O2 production in the range of μM. Nadolol was responsible of the release of 50 nM of NO and, more importantly, induced a 5 μM release of H_2_O_2_, which is probably leading to oxidative stress and cellular toxicity. Altogether, these data indicate that, even if they do not cross the BBB and do not accumulate in brain tissues, both atenolol and nadolol are able to induce central toxicity.

Of course, our study is limited by the use of murine *ex vivo* models which do not reflect the exact physiological conditions observed *in vivo*. A similar approach (i.e., NO/H_2_O_2_ real-time measurements) could be performed *in vivo* in different brain regions in mice in response to an oral load of beta-blockers (Fournel et al., 2017).

To conclude, our study brings new elements to decipher the mode of action (and the potential related side effects) of beta-blockers in the brain. The mode of communication through endothelial cells and production of NO/H_2_O_2_ that does not require the entry of a beta-blocker molecule into the brain has to be considered when using such molecule.

## Author Contributions

All authors have designed the experiments and wrote the manuscript. AA had performed the experiments.

## Conflict of Interest Statement

The authors declare that the research was conducted in the absence of any commercial or financial relationships that could be construed as a potential conflict of interest.
